# The need for health system strengthening in the wake of natural disasters: Lessons from the 2023 Türkiye–Syria earthquake

**DOI:** 10.1002/puh2.131

**Published:** 2023-10-21

**Authors:** Emery Manirambona, Joseph Christian Obnial, Shuaibu Saidu Musa, Adriana Viola Miranda, Usman Abubakar Haruna, Deborah Oluwaseun Shomuyiwa

**Affiliations:** ^1^ College of Medicine and Health Sciences University of Rwanda Kigali Rwanda; ^2^ Faculty of Medicine and Surgery University of Santo Tomas Manila Philippines; ^3^ Department of Nursing Science Ahmadu Bello University Zaria Nigeria; ^4^ Global Health Focus (GHF) Asia Bandung Indonesia; ^5^ Department of Biomedical Sciences Nazarbayev University School of Medicine Astana Kazakhstan; ^6^ Faculty of Pharmacy University of Lagos Lagos Nigeria

**Keywords:** disaster reduction, disasters, earthquakes, health system strengthening, natural disasters, resilience, Türkiye–Syria earthquake

## Abstract

The 2023 Türkiye–Syria earthquake was reported as the largest earthquake of Mw7.8, resulting in over 50,783 and 7259 deaths in Turkey and Syria, respectively. It has also damaged numerous residential buildings and other essential infrastructures, thus rendering more than 850,000 children and 356,000 pregnant women homeless, forcing them into displacement and its dire consequences, such as inadequate temporary shelters, a lack of access to safe drinking water, sanitation and hygiene (WASH), necessary for disease prevention, health promotion and maintenance. The disaster has disproportionately affected the Syrian refugee community in Turkey as it has fuelled disparities and discrimination, exacerbating the response to the disaster and forcing refugees to return to Syria due to dire living conditions. Minimizing the effects of the disaster on the communities is therefore essential. There is a need to strengthen health system resilience and emergency response to natural disasters to reduce and prevent the aftermath. Disaster preparedness plans should include regulations that ensure that local buildings and infrastructure are disaster‐resistant. Furthermore, it is vital to highlight the importance of funding and appropriate resource allocation for disaster risk reduction. These include improving plans and logistics for recovery efforts, adequate preparation of temporary shelters and evacuation centres and allocating necessities such as food and water. Investment in proper search and rescue response, a special workforce for response and the rebuilding of important infrastructure are crucial. Finally, response to disasters must be inclusive and prioritize vulnerable populations, such as children, the aged women and refugees.

## INTRODUCTION

Natural disasters are global health issues. Their consequences add on to the burden of health systems and are detrimental to physical health and mental health, as well as the general well‐being of the affected individuals and nations. The health impacts of natural disasters are multitudinous, including but not limited to chronic disease emergencies, the spread of communicable diseases, hygiene issues, mental health challenges, damage to healthcare infrastructure, food shortages and injuries. These impacts adversely affect communities living in affected areas, leaving them in precarious conditions as was the case with the recent disaster in Turkey.

Turkey experiences natural disasters that include floods, wildfires, landslides and earthquakes owing to its geomorphological structure and geographical location. Earthquakes are the most common disasters in the country, with the minimum of five magnitude earthquakes each year, thus holding the third position worldwide with regard to earthquake‐related casualties [1]. Considering the adverse implications of the earthquakes to health on the Turkish and Syrian communities, it is crucial to minimize their effects on the communities. This shows the need for a strong resilient response to natural disasters in order to better reduce and prevent the aftermath. Hence, this article explores the impact of the 2023 Türkiye–Syria earthquake on national public health and presents essential recommendations for improved pandemic preparedness, offering valuable insights for health policymakers.

## IMPACT AND CHALLENGES OF THE 2023 TÜRKIYE–SYRIA EARTHQUAKE

The recent earthquake struck Gaziantep, Turkey, on February 6, 2023, near the convergence of the Pazarcik and Amanos segments of the East Anatolian Fault (EAF), specifically at the Kahramanmaras Triple Junction (KMTJ). This city is highly susceptible to earthquakes due to its location within a tectonically active region, influenced by the movement of plates, such as Anatolia, Arabia, Africa and Eurasia. The ongoing pressure exerted by the Arabia and Africa plates against the Anatolia plate, in their lateral sliding and collision, has created a significant strike–slip fault [2]. This geological set‐up led to the most powerful earthquake ever recorded in Turkey, measuring Mw7.8 on the Richter Scale [2]. Such magnitude is deeply concerning, as major earthquakes of this scale can flatten well‐constructed infrastructure and impact areas extending hundreds of kilometres from the epicentre.

The aforementioned earthquake stands as one of the most devastating disasters of the 21st century, profoundly affecting communities in both Turkey and Syria. The toll in terms of injuries was staggering, with over 50,783 lives lost in Turkey and 7259 lives lost in Syria [3]. Additionally, it wreaked havoc on critical infrastructure, including numerous residential buildings, resulting in widespread homelessness and the forced displacement of more than 850,000 children and 356,000 pregnant women across both countries [4]. This disaster, along with its subsequent aftershocks, has significantly exacerbated the vulnerability of children and pregnant women in the region.

Furthering the above impact, the post‐disaster period has been marked by significant basic needs that have been unmet in temporary shelters, with a staggering gap concerning access to life‐saving water, sanitation and hygiene (WASH), increasing the risk of infectious diseases such as respiratory viral infections and food and waterborne diseases [5]. This is further compounded by the harsh weather during the winter season, which can further contribute to weakened immune systems. In addition, food shortage has been imminent in both countries [6], particularly Syria, where the existing conflict has made access to the region more difficult [7]. A shortage of temporary shelters has also been reported even as far as 3 weeks after the earthquake. Some of these shelters are located near damaged buildings, posing safety risks for families, particularly children and persons with disabilities [8].

In Syria, the earthquake has compounded the devastating effects of the current conflict, further aggravating the precarious conditions, such as lack of access to water, proper hygiene and electricity and freezing weather. With the earthquake predominantly affecting a region occupied mainly by opposition forces, the Syrian government compelled any aid to pass through government‐controlled areas, hampering the delivery of much needed food and shelter to affected citizens [7]. This is critical because the affected region has been hosting refugees and internally displaced persons who depend on humanitarian aid and barely have access to basic needs and healthcare services.

The Syrian refugee community in Turkey has disproportionately been affected. They did not only face uneven treatment and discrimination in terms of health emergency interventions, but Syrian refugees were also removed from the camps where they had resided with temporary protection status in order to bring in Turkish citizens [3]. Therefore, it is unsurprising that some Syrian refugees were forced to return to Syria because of unbearable dire conditions caused by the earthquake and uneven treatment [3]. This is even more striking because the fate awaiting the refugees returning in Syria is much more uncertain.

Natural disasters such as the recent earthquake cause significant indirect damage to public health. The earthquake devastated healthcare facilities, leading to a significant shortage of necessary medical infrastructure. Numerous healthcare workers can be presumed to have lost their lives in the disaster, contributing to the loss of human resources that could have been allocated to triage and emergency medical services. Other effects of disasters, such as psychological issues and trauma, are also significant among those affected by the earthquake, with survivors experiencing mental health issues such as anxiety and post‐traumatic stress disorder [9]. This is even more pronounced in Syria, where humanitarian crises have been ongoing because of the conflict.

The extent of damage wrought by the disaster and the poor disaster response of the Turkish government may indicate that previous disaster preparedness strategies were not fully implemented, following lessons learned from the 1999 İzmit Earthquake. An earthquake of such a scale has been predicted since the 1999 earthquake, and the government has been urged to reinforce the country's building codes to ensure that its buildings can withstand such calamities in the future. However, the massive amount of collapsed buildings may indicate that these regulations have not been adequately enforced [10, 11]. In fact, in the aftermath of the 1999 earthquake, Turkey instituted ‘earthquake solidarity taxes’ to fund earthquake‐resistant infrastructure, but the outcomes of this tax remain to be seen [11]. More importantly, the Turkish government instituted zoning amnesties in 2018, which allowed the construction of buildings without regard to building codes in exchange for a fee [10, 11]. This was reported by experts to be a major factor for the immense amount of building collapse. Despite a significant workforce, search and rescue have also been viewed as delayed and sluggish [11]. In Syria, the recent conflict has prevented large‐scale rescue efforts, with survivors reportedly taking the matter into their own hands [9]. The above‐mentioned challenges are summarized in Table 1.

**TABLE 1:** Challenges and recommendations.

Challenges	Recommendations
Difficulties with the initial response to the disaster/lack of strong preparedness	Establish a strong disaster emergency committee Enhance disaster prevention and response capabilities Foster international cooperation in disaster aftermath^a^
Difficulties for delivering aid in conflicts areas	Open additional operational aid corridors^a^
Lack/Delay in search and rescue	Train and deploy specialized response teams^a^
Difficult task of disposing of hundreds of millions of tonnes of rubble	Train specialized response teams and provide necessary equipment
Gap in access to life‐saving WASH, food shortage, lack of shelters	Improving plans and logistics of allocating necessities such as WASH, food and shelters^a^
Destruction of public, health facilities	Reinforce the country's building codes/rebuild health infrastructure^a^
Lack of health equipment	Equip healthcare systems Provide medicines and relief items^a^
Psychological issue/PTSD	Prioritize psychological first aid post‐disaster
Discrimination against refugees	Consider refugees in disaster response – promote inclusive aid distribution^a^

Abbreviations: PTSD, post‐traumatic stress disorder; WASH, water, sanitation and hygiene.

^a^ International cooperation in the aftermath of disasters.

## RECOMMENDATIONS

The challenges presented by earthquakes in Syria and Turkey illustrated the need to build a strong health system able to respond to emergency interventions. Strengthening health systems must include a comprehensive and integrative approach to disaster risk reduction in all of its phases, from mitigation and preparedness to response and recovery. Disaster preparedness plans should include regulations that ensure that local buildings and infrastructure are disaster‐ready. In the case of Turkey, building regulations must be thoroughly enforced to reduce the impact of another high‐magnitude earthquake. Furthermore, it is vital to highlight the importance of funding and appropriate resource allocation for disaster risk reduction. These include improving plans and logistics for recovery efforts, adequate preparation of temporary shelters and evacuation centres and allocating necessities such as food and water. Investment in proper search and rescue response, a special workforce for response, and the rebuilding of important infrastructure are crucial (Table 1).

Building health system resilience and responsiveness is also essential to reducing the impact of disasters and empowering communities to prepare for, respond to and recover from disasters. Designing community‐specific programmes tailored to their needs and resources will empower ownership and optimal participation in their preparedness and recovery efforts. In the aftermath of a disaster, psychological first aid must also be given priority to aid the community in coping with recent traumatic events [9].

The deadliest effects of the earthquake highlight the need for international cooperation in the aftermath of disasters. Even with appropriate disaster preparedness, it would still be difficult for both countries to recover independently. Regional organizations such as the Arab League and the European Union should work in harmony with the United Nations and other international nongovernmental organizations (INGOs) in building and equipping the destroyed health system and providing medicines and relief items to the country. Extending support to include technical expertise and resources necessary for rescue and recovery efforts is also essential to mitigate the impact, as the crisis can overwhelm the nation's government. Notably, any effort to tackle devastating effects of the quakes must consider refugees, and any sort of discrimination should be discouraged. Collaboration to ensure that aid and resources are distributed effectively and efficiently is vital. This is especially seen in the case of Syria, where international actors must find common ground to cease hostilities to ensure that aid reaches those in need. In the end, with the threat of more aftershocks and future disasters, it is imperative for all parties, national and international, to act now to avoid preventable loss of lives, now and in the future.

## CONCLUSION

The devastating effects of the 2023 Türkiye–Syria earthquake underscore the critical importance of bolstering health system resilience and responsiveness to mitigate the impact of disasters on communities. It is imperative for each country to establish and adequately fund a Disaster Emergency Committee to enhance disaster prevention and emergency service development. Given the unprecedented scale of such disasters, there is an urgent need for comprehensive plans and logistical strategies for the swift allocation of essentials like WASH, shelter and food as part of emergency response efforts.

## AUTHOR CONTRIBUTIONS

*Conceptualization; project administration; data curation; investigation; writing – original draft; review and editing*: Emery Manirambona. *Data curation; investigation; writing – original draft; writing – review and editing*: Joseph Christian Obnial, Shuaibu Saidu Musa, Adriana Viola Miranda, Usman Abubakar Haruna and Deborah Oluwaseun Shomuyiwa.

## CONFLICT OF INTEREST STATEMENT

Emery Manirambona, Joseph Christian Obnial, Shuaibu Saidu Musa, Adriana Viola Miranda, Usman Abubakar Haruna and Deborah Oluwaseun Shomuyiwa are Youth Editorial Board members of the journal. They were excluded and blinded from all stages of the peer review of this manuscript.

## ETHICS STATEMENT

Ethical clearance was not required because we did not deal with human or animal data; thus, informed consents were not needed.

## Data Availability

The sources of the data used are available in the reference section of the manuscript.
